# Changes in Serum *Strongylus Vulgaris*-Specific Antibody Concentrations in Response to Anthelmintic Treatment of Experimentally Infected Foals

**DOI:** 10.3389/fvets.2015.00017

**Published:** 2015-07-01

**Authors:** Martin Krarup Nielsen, Jessica Scare, Holli Sullivan Gravatte, Jennifer Lynn Bellaw, Julio C. Prado, Craig Robert Reinemeyer

**Affiliations:** ^1^M.H. Gluck Equine Research Center, Department of Veterinary Science, University of Kentucky, Lexington, KY, USA; ^2^East Tennessee Clinical Research, Inc., Rockwood, TN, USA

**Keywords:** *Strongylus vulgaris*, diagnosis, larval migration, ivermectin, ELISA

## Abstract

*Strongylus vulgaris* is the most pathogenic nematode parasite of horses. Its extensive migration in the mesenteric blood vessels can lead to life-threatening intestinal infarctions. Recent work has shown that this parasite is still identified among managed horse populations. A serum enzyme-linked immunosorbent assay (ELISA) has been developed for the detection of migrating larvae of *S. vulgaris*. Previous work has documented an increase in ELISA values following larvicidal treatment with ivermectin and suggested that the target parasite antigen is primarily produced by the later larval stages. The aim of this study was to experimentally inoculate cohorts of foals with *S. vulgaris*, and then compare ELISA responses to early or later ivermectin treatments. Fifteen foals were held in confinement and infected orally with ~25 *S. vulgaris* third-stage larvae on Days 0, 7, 14, and 21. Foals were weaned on Day 43 and turned out to a pasture not previously grazed by horses. Foals remained at pasture continuously until the study was terminated on Day 196. On Day 55, foals were randomly allocated to three treatment groups of five each. Group 1 received ivermectin on Day 56, Group 2 received ivermectin on Day 112, and Group 3 foals served as untreated controls. Serum and fecal samples were collected at 28-day intervals throughout the study. Serum samples were analyzed with the *S. vulgaris*-specific ELISA and fecal samples were processed for fecal egg counting. The ELISA values of Group 1 foals were significantly lower than Groups 2 or 3 on Days 140–196. Both treated groups exhibited increased ELISA values following ivermectin treatment. Results indicate that the target diagnostic antigen is produced throughout the course of arterial infection with *S. vulgaris*, but that an early ivermectin treatment can reduce the cumulative antigen produced over the course of an infection.

## Introduction

*Strongylus vulgaris* is considered to be the most pathogenic helminth parasite of horses due to the extensive migration of its larval stages. The predilection site for migration is the cranial mesenteric artery (CMA) and its major branches. Upon infection, exsheathed third-stage larvae penetrate the large intestinal mucosa, where they molt to the fourth larval stage (L4) within the first 5 days. The L4s then migrate proximally toward the CMA, where they arrive about 11–14 days post infection (PI). In the CMA, fourth-stage larvae (L4) molt to the immature adult stage (L5) at about 90 days PI, and migration back to the large intestine begins around Day 120 PI (1). Migrating larvae cause fibrinous endarteritis with pronounced thrombosis, formation of aneurysms, and other pathologic alterations of the vessel structures (2). Thrombo-embolism caused by L4s and immature adults has been associated with a painful and often fatal colic syndrome, characterized by ischemia and non-strangulating infarction of intestinal segments (3–5).

Historically, *S. vulgaris* was prevalent in virtually all grazing horses (6–8). However, this pattern changed during the 1980s, when a significant decline was attributed to the intensive anthelmintic treatment regimens typically implemented for horse populations (9, 10). Notwithstanding, subsequent surveys of managed horses across the world have documented that *S. vulgaris* continues to be encountered on a regular basis (11–13). One recent study found *S. vulgaris* DNA in fecal samples from regularly dewormed thoroughbred mares in Central Kentucky (14). Another recent study conducted in Denmark has even documented a higher occurrence of *S. vulgaris* on farms using a widely recommended parasite control strategy in which treatment decisions are based on pre-treatment fecal egg count levels (15). Further, a recent retrospective case-control study conducted among referred Danish equine patients documented a significant association between non-strangulating intestinal infarctions and serological evidence of *S. vulgaris* infection (5). Taken together, these reports emphasize the need for reliable diagnostic assays to detect *S. vulgaris* infections in managed horses.

Recently, a serum enzyme-linked immunosorbent assay (ELISA) was developed and validated to detect migrating *S. vulgaris* larvae in the bloodstream of horses (16). The assay measures host IgG(T) antibodies against a recombinant *S. vulgaris* SXP antigen, *Strongylus vulgaris* serine-X-proline (SvSXP), and returns a diagnostic sensitivity of 73.3%, a specificity of 81.0%, and a statistically significant correlation with the numbers of migrating larvae in the mesenteric arteries (16). This assay has been further evaluated and characterized in recent studies. One study performed with naturally infected foals documented the presence of maternal antibodies during the first weeks of life, and found that foals became ELISA-positive between 3 and 5 months of age (17). A second study evaluated the effect of ivermectin treatment in ELISA-positive juvenile horses and illustrated an initial increase in ELISA values following treatment, followed by a decline which was complete after 5 months (18). In the same study, an untreated control group had a significant increase of ELISA values after approximately 5 months of natural exposure to *S. vulgaris* infection at pasture. Taken together, these two studies illustrate that a positive ELISA result represents either current or recent infection with *S. vulgaris* within the preceding 5 months. Further, it appears that the SvSXP antigen may be produced primarily by later larval stages because ELISA values increase markedly about 5 months after first exposure to infection.

The purpose of this study was to test the hypothesis that SvSXP is primarily produced by the immature L5 stages present in the CMA after about 90 days PI. The hypothesis was tested by experimentally infecting cohorts of foals with *S. vulgaris* and then comparing SvSXP ELISA responses after early (<90 days) and later (>90 days) larvicidal treatments with ivermectin.

## Materials and Methods

This was a controlled, randomized, blinded prospective clinical study conducted at a single site between July 2, 2014 and January 14, 2015. The study was reviewed and approved by East Tennessee Clinical Research’s Institutional Animal Care and Use Committee (Application No. ETCR-13-0136, approved 22MAY14).

### Foals

Fifteen nursing mares with foals-at-side were acquired by East Tennessee Clinical Research in Rockwood, TN, USA. Prior to the onset of the study, mares and foals were held in confinement, and offered a commercial equine concentrate (11% protein) in quantities totaling ~0.5% of the mare’s body weight, divided into similar portions offered twice daily. Grass hay was offered twice daily in quantities totaling ~2.0% of the mare’s body weight. Water from a commercial utility was provided *ad libitum*.

Approximately 1–4 weeks prior to initiation of the trial, all mares and foals received a larvicidal anthelmintic regimen of fenbendazole (10 mg/kg once daily for five consecutive days; Panacur Powerpak, Merck Animal Health, Millsboro, DE, USA). Fecal egg counts were performed to confirm that the treatment worked.

The 15 foals enrolled in the study were of various light saddle breeds, including Appaloosa, Quarter Horse, Tennessee Walking Horse, and Thoroughbred, and were born between April 24 and June, 2014. Nine were females, and six were intact males. All underwent a physical examination and were found healthy prior to the onset of the study.

All foals were inoculated orally with ~25 infective third-stage larvae (L3) of *S. vulgaris* on Days 0, 7, 14, and 21. This infective dose has been successfully used in a previous study (19). On Day 43 (August 14, 2014), foals were separated from their dams and turned out on a pasture not previously grazed by horses, where they remained for the duration of the study. After turnout, foals were offered a commercial equine concentrate (11% protein) in quantities totaling ~0.25% of body weight once daily. Pasture forage was the primary source of fiber and energy, but supplemental grass hay was available *ad libitum* whenever pasture forage was inadequate for maintenance. Water was available *ad libitum* via automatic, frost-free hydrants.

Foals were ranked according to their arrival date. Each three consecutively ranked foals comprised a block, and foals were allocated randomly from each block to the three treatment group. Foals assigned to Groups 1 and 2 were treated orally with ivermectin (200 μg/kg; Zimecterin, Merial Ltd., Duluth, GA, USA) on Days 56 and 112, respectively. Foals assigned to Group 3 served as untreated controls. Body weights were measured with a certified scale, and fecal and serum samples were collected from each enrolled foal on Day 0 (July 2, 2014) and repeated thereafter at 28-day intervals until termination of the study on Day 196 (January 14, 2015).

Randomization of horses to study groups and preparation of individual doses of ivermectin were completed by personnel who determined no outcome measures for the duration of the trial. Administration of the anthelmintic was the responsibility of employees who remained masked to treatment assignments. Records of treatment assignments were sequestered away from masked personnel to prevent inadvertent unblinding.

### Preparation and administration of *S. vulgaris* infective larvae

Feces were collected from two mares known to have patent *S. vulgaris* infections. The mares were residents of the parasitology research herd of the University of Kentucky (20). Individual 20 g fecal samples were collected fresh and mixed with equal volumes of vermiculite (Infinity Fertilizers, Inc., Milan, IL, USA) and moistened with tap water until pliable. Fecal mixtures were then suspended in cheesecloth over 10 mL of tap water in humidity chambers as described by Henriksen and Korsholm (21). These chambers were incubated at ~25°C for 14 days and moistened as necessary. The fecal mixtures were then sedimented for 24 h in Baermann apparatuses consisting of wine glasses containing reservoirs in their stems.

After 24 h, the fecal mixtures were removed from the Baermann apparatus and the entire contents were centrifuged in multiple 50 mL tubes at ~200 g for 10 min. The pellets in each tube were then consolidated into a single 15 mL tube per sample and stored at 25°C until examination. For examination and enumeration of larvae present, each pellet was re-suspended and transferred to nematode counting chambers (Chalex Corp., Ketchum, ID, USA). Nematode slides were examined at 100× magnification. Approximate numbers of *S. vulgaris* third-stage larvae were recorded, and other observed species and stages were noted as identified according to published criteria (22). Larvae were transferred to 15 mL tubes, filled with tap water, covered with parafilm, and capped before shipment to the testing facility (East Tennessee Clinical Research).

On the days of inoculation, larval counts were conducted, and volumes of inoculum were prepared to contain ~25 infective L3s of *S. vulgaris*. A separate inoculum was prepared for each foal; larvae were transported to the animal facility in individual; labeled 15 mL polypropylene tubes. Larvae were administered orally to each foal using a separate syringe fitted with polyethylene tubing. After oral administration of the inoculum, the transport tube was rinsed with ~5 mL of tap water, which was aspirated into the original inoculation syringe, and administered to the recipient foal in an identical fashion.

### Fecal egg counts

Fecal egg counts were performed in triplicate for each enrolled foal at every scheduled time point, using the mini-FLOTAC egg counting technique with a detection limit of 5 EPG (23). A saturated glucose-salt solution was used as flotation medium (specific gravity: 1.26).

### SVSXP serum ELISA

Serum samples were collected from all enrolled horses at regular, 28-day intervals throughout the study. An indirect ELISA using recombinant SvSXP protein as antigen was implemented as described previously, with duplicate measurements of each sample (16). Serum samples were diluted 1:50 and horseradish peroxidase (HRP)-conjugated goat anti-horse IgG(T) (Bethyl Laboratories, Inc., Montgomery, TX, USA) was used as a secondary antibody at a dilution of 1:40,000. The result was reported as the normalized value, percentage of a positive control (PP), in order to reduce inter-assay variability (16).

### *Strongylus vulgaris*-specific PCR

At termination of the study, strongyle eggs were recovered from fecal samples collected on Day 196. Strongyle eggs were analyzed for the presence of *S. vulgaris* DNA using a species-specific real-time PCR assay described previously (24). All positive reactions were reanalyzed to rule out false amplification. Results were reported as the cycle of threshold (Ct) for a positive PCR amplification, and no amplification in case of PCR negative tests.

### Coproculture for presence of *strongylus vulgaris* larvae

The samples collected on Day 196 were also coprocultured to test for the presence of patent *S. vulgaris* infection. The coproculture procedure was similar to the one described previously for preparation of infective inocula. Diagnostic coprocultures utilized 10 g of feces from each sample, and the entire sediment was collected from each Baermann apparatus after 48 h of sedimentation. Larvae were identified using published morphological criteria (22).

### Statistical analyses

All analyses were carried out using SAS software (version 9.3, SAS Institute). Mixed linear models were constructed for analyzing the relationship between serum ELISA values and anthelmintic treatments (group) and time point and the interaction term between the two. The “mixed” procedure was used with repeated measures and “foal ID” as random effect. “Group,” “gender,” “age,” and “time point” were kept as class variables, while all other variables were considered continuous. The influence of all measured parameters and interactions was evaluated using traditional forward and backward elimination of variables. All variables with *p*-values of 0.20 or below were kept in the model. Variables were log-transformed to achieve normal distribution, where appropriate. Whenever the variables “time point,” “group,” or the interaction term “time point by group” were found significant, a “least square means” analysis was used for a Tukey’s pairwise comparison. Results were interpreted at the 0.05 significance level.

## Results

**Figure 1:**
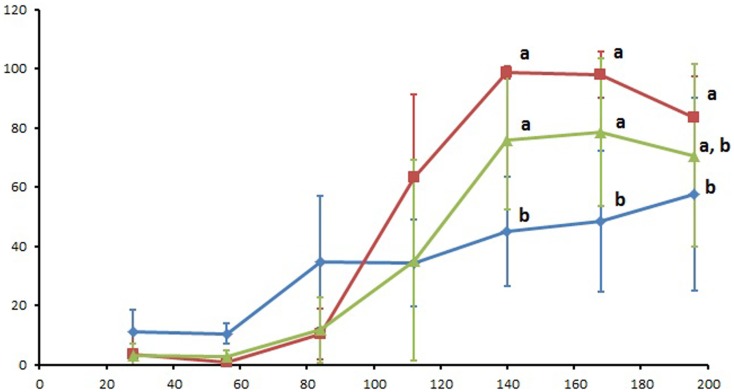
**Serum SvSXP ELISA values presented as a percentage of a positive control over the course of the 196 study days**. Blue diamonds: group 1 (treated with ivermectin on Day 56), red squares: group 2 (treated with ivermectin on Day 112), and green triangles: group 3 (no treatment control). Different letters designate statistically significant differences (*p* < 0.05) between groups at the given time points. Error bars represent 95% confidence intervals.

The SvSXP ELISA values measured over the course of the study are presented in Figure 1. All individual ELISA values are included as supplementary material. Statistical differences between treatment groups were observed from Day 140 onward, with Group 1 having significantly lower mean ELISA values (*p* < 0.05) than the other two groups. Results of the Tukey’s pairwise comparison of time points within each treatment group are presented in Table 1. Whereas Group 1 had a steady increase of ELISA values over the course of the study, the two other groups exhibited increases between Days 84 and 140, after which they both reached a plateau.

**Table 1:** ***p*-Values generated in the Tukey’s pairwise comparison analysis of the SvSXP ELISA results obtained at different time points within each treatment group in the study**.

Day	28	54	84	112	140	168	196
**Group 1: Ivermectin treatment on day 56**							
28	–						
54	1.0000	–					
84	0.1527	**0.0042**	–				
112	0.8788	0.1747	0.9999	–			
140	0.0841	**0.0017**	1.0000	0.9990	–		
168	0.1721	**0.0051**	1.0000	1.0000	1.0000	–	
196	**0.0065**	**<0.0001**	1.0000	0.8713	1.0000	1.0000	–
**Group 2: Ivermectin treatment on day 112**							
28	–						
54	1.0000	–					
84	0.9999	0.9922	–				
112	**<0.0001**	**<0.0001**	**<0.0001**	–			
140	**<0.0001**	**<0.0001**	**<0.0001**	**<0.0001**	–		
168	**<0.0001**	**<0.0001**	**<0.0001**	**<0.0001**	1.0000	–	
196	**<0.0001**	**<0.0001**	**<0.0001**	**0.0255**	0.3337	0.4014	–
**Group 3: No treatment**							
28	–						
54	1.0000	–					
84	0.9940	0.9816	–				
112	**<0.0001**	**<0.0001**	**0.0027**	–			
140	**<0.0001**	**<0.0001**	**<0.0001**	**<0.0001**	–		
168	**<0.0001**	**<0.0001**	**<0.0001**	**<0.0001**	1.0000	–	
196	**<0.0001**	**<0.0001**	**<0.0001**	**<0.0001**	1.0000	0.9959	–

*Significant differences are highlighted in bold*.

**Figure 2:**
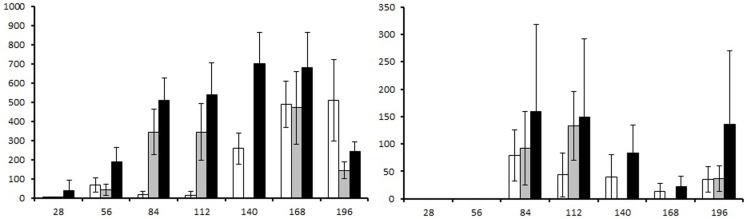
**Fecal egg counts presented as eggs per gram (EPG) of feces generated over course of the study**. Left graph: strongyle fecal egg count, right graph: ascarid fecal egg count. White columns: group 1 (ivermectin treatment on Day 56), gray columns: group 2 (ivermectin treatment on Day 112), and black columns: group 3 (no treatment control). Error bars represent 95% confidence intervals.

Strongyle and ascarid egg count data are presented in Figure 2. The two ivermectin treatments applied in Groups 1 and 2 reduced strongyle egg counts by 73 and 100%, respectively, by the next sampling time point 28 days later. For the ascarid egg counts, no reduction was observed after the Day 56 ivermectin treatment in Group 1, whereas the Day 112 treatment in Group 2 reduced the counts by 100%.

The coproculture and PCR results for detecting patent *S. vulgaris* infections at trial termination are presented in Table 2.

**Table 2:** **Results of diagnostic work performed for presence patent *Strongylus vulgaris* infection at Day 196**.

	PCR^a^	Coproculture^b^
**Group 1: Ivermectin treatment on day 56**		
703	–	4
707	–	0
708	–	0
710	–	0
757	41.46	0
**Group 2: Ivermectin treatment on day 112**		
702	–	0
709	30.26	0
730	38.52	0
733	36.62	0
750	34.53	2
**Group 3: No treatment**		
705	–	0
731	37.16	0
732	33.61	0
753	28.30	8
754	34.90	4

*A dash (–) indicates no PCR amplification*.

*^a^Results of a *S. vulgaris*-specific polymerase chain reaction assay reported as the mean cycle of threshold*.

*^b^The number of *S. vulgaris* third-stage larvae identified in the entire culture*.

## Discussion

This study generated useful new information about elaboration of *S. vulgaris*-specific antibodies in response to ivermectin treatment of an established infection with migrating larvae. The group treated early in the infection (Day 56) had a markedly different ELISA response than control horses or the group treated with ivermectin on Day 112. While this pattern could suggest differences in SvSXP antigen production by L4 and L5 larval stages, increased antibody levels in all groups after Day 56 (Figure 1) are evidence that antigen is undoubtedly produced by the L4 stages. It is feasible that the more pronounced ELISA responses in Group 2 and 3 foals was attributable to the continuous and cumulative production of SvSXP over the course of several months. Previous work has illustrated that this antigen is highly immunogenic (16), so continuous release is likely to stimulate a more pronounced antibody response. The abrupt increases observed after Day 84 in Groups 2 and 3 closely resemble the response observed in a cohort of foals exposed to natural infection with *S. vulgaris* (17). Thus, if left untreated, foals exposed to *S. vulgaris* infection will undergo a marked seroconversion, which can be interpreted as highly indicative of an active arterial infection.

It is worth noting that ELISA values increased following both ivermectin treatments and that this difference was statistically significant in Group 1 (Table 1). This finding is consistent with observations in a study evaluating ivermectin treatment of naturally infected juvenile horses (18), and we have hypothesized that this increased antibody production is due to dying worms which exacerbate interactions with the immune system. It has been shown that arterial stages of *S. vulgaris* die over an interval of 4 weeks following ivermectin treatment (25, 26). The nematocidal activity of ivermectin against migrating larvae is mediated through host immune mechanisms, and dead larvae are eventually translocated from the vessel lumen to the subendothelial tunica media of the arteries (26). Thus, it appears plausible that an increased production of antibodies to the SvSXP antigen can occur during a 4-week period following ivermectin treatment.

Table 2 suggests that ivermectin treatment was not 100% efficacious against migrating stages of *S. vulgaris* in the current study, and that the efficacy was perhaps even less against the later stages of infection (L5). Ivermectin is reportedly >98% efficacious against migrating larvae of *S. vulgaris* (25, 27, 28), so historical evidence indicates that a few larvae can survive. However, these studies all evaluated the efficacy against 56-day-old experimental infections whereas no published studies appear to have evaluated ivermectin efficacy against *S. vulgaris* at ~112 days post inoculation. It is worth noting that fecal samples from several horses tested PCR positive for *S. vulgaris* 5 months following ivermectin treatment in a recent study (18). The fecal egg count reduction observed after ivermectin administration in group 1 was less than desired, but it should be kept in mind that pre-treatment egg count levels were very low and that eggs ingested by coprophagy could constitute a plausible source of error post-treatment in this age group. Therefore, it remains possible that a portion of *S. vulgaris* L5 larvae can survive ivermectin treatment. However, this should be evaluated in appropriate research studies before any conclusions can be drawn. It is also worth noticing that the coproculture and the PCR were not always in perfect agreement. In a previous study, we have reported a kappa value of 0.5405 between the two tests (29). One possible explanation is that we routinely examine the entire sediment of larvae for presence of *S. vulgaris*, and this increases the diagnostic sensitivity to a level comparable to the PCR. Eggs of *S. vulgaris* are unlikely to be evenly distributed over the fecal matter, so some of the discrepancy between the two procedures could simply be due to differences between subsamples.

The survival of some larvae after ivermectin treatment would explain the steady increase of antibodies observed in Group 1 during the 5 months following treatment (Figure 1). Further, antibody levels of the other ivermectin-treated horses in Group 2 did not differ from the untreated control group (Group 3) at any of the seven time points. Again, one possible explanation is that a portion of *S. vulgaris* larvae survived treatment and continued their production of SvSXP. Further, we know from a recent study that ELISA values do not decrease until about 3–4 months following ivermectin treatment (18). In the present study, this means that a significant reduction was not likely to occur before the termination of the study. Further, since the prepatent period of *S. vulgaris* is about 6 months, the large majority of larvae should have left the vascular development site during the final months of the study. Perhaps, this exodus can explain the decline of ELISA values observed in Groups 2 and 3 during the last three time points. In other words, even the untreated control group experienced a reduction of arterial larvae during this time, so any treatment-induced effects on ELISA values in Group 2 would not be markedly different.

In summary, this study provided documentation that the SvSXP antigen is apparently produced throughout the course of arterial infection with *S. vulgaris*. Thus, our hypothesis that the antigen is only produced by later migrating stages can be rejected. However, an early treatment interruption will significantly reduce the concentration of serum SvSXP-specific antibodies in subsequent months. Further, this study also suggested that ivermectin efficacy against migrating *S. vulgaris* larvae may be <100%, but this has yet to be evaluated in appropriate studies.

## Author Contributions

CRR and MKN developed the study protocol and oversaw all procedures. JCP supervised and implemented the clinical elements of the study. JS, HSG, and JLB contributed substantially to acquisition and interpretation of the data. MKN drafted the manuscript with contribution from all authors. The manuscript was critically reviewed and the final version approved by all authors.

## Conflict of Interest Statement

The authors declare that the research was conducted in the absence of any commercial or financial relationships that could be construed as a potential conflict of interest.
